# Translation, Cultural Adaptation, and Reliability and Validity Testing of a Chinese Version of the Freezing of Gait Questionnaire (FOGQ-CH)

**DOI:** 10.3389/fneur.2021.760398

**Published:** 2021-11-23

**Authors:** Ping Tao, Xuerong Shao, Jie Zhuang, Zhen Wang, Yuchen Dong, Xia Shen, Yunjie Guo, Xiaoyi Shu, Hong Wang, Yuanhong Xu, Zhenlan Li, Roger Adams, Jia Han

**Affiliations:** ^1^School of Kinesiology, Shanghai University of Sport, Shanghai, China; ^2^School of Medicine, Jinhua Polytechnic, Jinhua, China; ^3^School of Martial Arts, Shanghai University of Sport, Shanghai, China; ^4^School of Medicine, Tongji University, Shanghai, China; ^5^Shanghai YangZhi Rehabilitation Hospital (Shanghai Sunshine Rehabilitation Center), Tongji University School of Medicine, Shanghai, China; ^6^Department of Rehabilitation Medicine, Shenzhen Samii International Medical Center (The Fourth People's Hospital of Shenzhen), Shenzhen, China; ^7^College of Rehabilitation Science, Shanghai University of Medicine and Health Sciences, Shanghai, China; ^8^Rehabilitation Department, Affiliated Taihe Hospital of Hubei University of Medicine, Shiyan, China; ^9^Department of Rehabilitation Sciences, Ningbo College of Health Sciences, Ningbo, China; ^10^Research Institute for Sports and Exercise, University of Canberra, Canberra, ACT, Australia; ^11^Faculty of Health, Arts and Design, Swinburne University of Technology, Hawthorn, VIC, Australia

**Keywords:** freezing of gait, freezing of gait questionnaire, Parkinson's disease, fall, translation, reliability study, validity study

## Abstract

Freezing of gait is a disabling symptom with a complex episodic nature that is frequently experienced by people with Parkinson's disease (PD). Although China has the largest population with PD in the world, no Chinese version of the freezing of gait questionnaire (FOGQ), the instrument that has been most widely used to assess FOG, has yet been developed. This study aimed to translate and adapt the original version of FOGQ to create a Chinese version, the FOGQ-CH, then assess its reliability, calculate the Minimal Detectable Change (MDC) and investigate its validity. The forward-backwards translation model was adopted, and cultural adaptation included expert review and pretesting. For the reliability study, 31 Chinese native speaking patients with PD were assessed two times in a 7–10 days interval. Internal consistency and test-retest reliability of the FOGQ-CH were measured by Cronbach's alpha (Cα) and the Intraclass Correlation Coefficient (ICC). For the validity study, 34 native speakers of Chinese with PD were included. To explore the convergent validity, relationships between the FOGQ-CH and the Unified Parkinson's Disease Rating Scale Part II (UPDRS II) and Part III (UPDRS III), Timed Up and Go Test (TUGT), Timed Up and Go Test in cognitive task (TUGT-Cog), walking speed (10 MWT speed), and step length (10 MWT step length) in a 10-m Walk Test were tested. To explore predictive validity, the number of falls followed up for 6 months were assessed. The area under the ROC curve (AUC) was employed to test the capacity of FOGQ-CH to discriminate those with falls. From the reliability study, Cα = 0.823, ICC = 0.786. The MDC_0.90_ = 4.538. From the validity study, the FOGQ-CH showed moderate correlations with UPDRS II (*rho* = 0.560, *p* = 0.001), UPDRS III (*rho* = 0.451, *p* = 0.007), TUGT (*rho* = 0.556, *p* = 0.007), TUGT-Cog (*rho* = 0.557, *p* = 0.001), 10MWT-speed (*rho* = −0.478, *p* = 0.004), 10MWT-step length (*rho* = −0.419, *p* = 0.014), and the number of falls followed up for 6 months (*rho* = 0.356, *p* = 0.045). The AUC = 0.777 (*p* = 0.036) for predicting whether the participants will have multiple falls (two or more) in the following 6 months. The FOGQ-CH showed good reliability and validity for assessing Chinese native speaking patients with PD. In addition, the FOGQ-CH showed good efficacy for predicting multiple falls in the following 6 months.

## Introduction

Freezing of gait is one of the most common disabling symptoms in Parkinson's disease (PD), and it affects ~63% of patients with idiopathic PD (1). Freezing of gait (FOG) has been defined as the experience of sudden and, usually, brief episodes with the inability to make effective steps (2). During the FOG episode, patients report the feeling that their feet are glued to the floor (2). FOG can lead to falling, low quality of life, and dependence (3). In addition, FOG has been hard to assess objectively (4).

The Freezing of gait questionnaire (FOGQ) is a patient-reported outcome measure, which was developed by Giladi et al. (5). It has six questions and can easily be administered in clinics. To date, the FOGQ has been translated to different languages, including Swedish, Italian, Turkish, Spanish, Brazilian, German, and Czech (4, 6–11), and these versions have shown good validity and reliability. China has the largest population with PD globally, and it is estimated that the number of patients with PD in China will reach half of all patients with PD in the world by 2030 (12). However, there is, at present, no Chinese version of the FOGQ. Accordingly, one of the aims of the present study was to develop a Chinese translation version of the FOGQ and assess its reliability and validity. In addition, while the Minimal Detectable Change (MDC) of the FOGQ is important when interpreting the change of the scores following treatment, it has not been reported in previous studies.

Falling is a sign of poor balance that can bring serious physical damage to patients with PD (i.e., fractures) (13). The early presence of falling is a risk factor in patients with PD (1). FOG was one factor of falling (3, 14); however, it is undetermined if the Chinese version of FOGQ can be used for fall prediction in Chinese patients with PD.

We hypothesize that the test-retest reliability of the Chinese version of the FOGQ (FOGQ-CH) will be reliable enough to be on par with the Unified Parkinson' s Disease Rating Scale (UPDRS) Part II and Part III involving activities of daily living (ADL) and motor performance (15, 16), three mobility measurements include the Timed Up and Go Test (TUGT), the Cognitive dual-task Timed Up and Go Test (TUGT-Cog) (17), and the 10-m Walk Test (10MWT) (18). In addition, it is hypothesized that the FOGQ-CH will show good predictive validity in terms of the number of falls in Chinese patients with PD.

## Materials and Methods

The study was approved by the Committee for Ethics in Human Research at Shanghai University of Sport (Approval number: 102772021RT038). All patients signed informed consent before the data collection.

### Part 1: Translation and Cross-Cultural Adaptation

**Figure 1:**
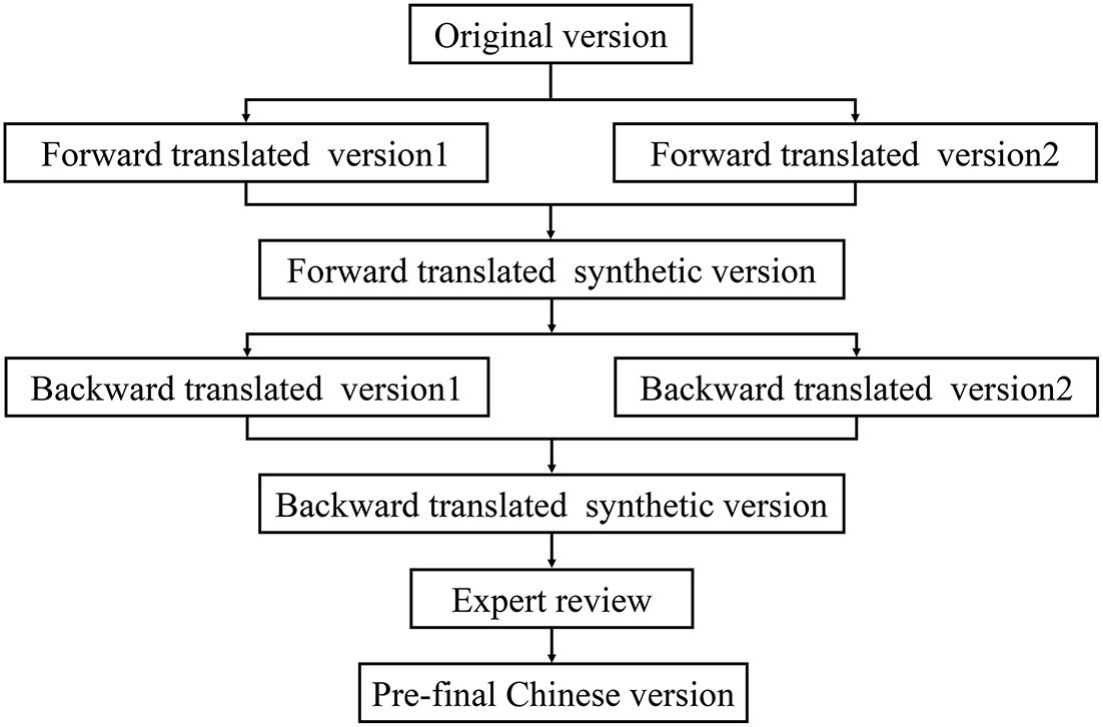
The procedure of the translation and cross-cultural adaptation of Chinese version of the Freezing gait questionnaire (FOGQ-CH).

The (FOGQ-CH) was translated by researchers based on a standard procedure of forward-backwards translation (19). The procedure is shown in Figure 1.

#### Forward Translation and Synthesis

Two native Chinese translators independently translated the English version to Chinese after obtaining permission from the corresponding author of the original English FOGQ. One translator was adept on research on Parkinson's disease, and another translator was a physiotherapist with a master's degree in Hongkong. Both were native Chinese speakers who had good English language skills. After two translators finished the forward translation, we got the forward translated version 1 and forward translated version 2. Any ambiguities and discrepancies in the two versions were discussed by our research team (which included a health science professor, a neural physiotherapist, and a doctor of Parkinson's disease). The research team then made a contrastive analysis and formed a forward translated synthetic version.

#### Backward Translation and Synthesis

After forward translation and synthesis, the forward translated synthetic Chinese version was independently translated back to English by another two translators who had never read the original version of FOGQ. Both of them had more than 2 years of study and life experience in Australia. One translator was a master of physiotherapy and another translator is a professor of English linguistics. After two translators finished the backward translation, we got the backward translated version 1 and forward translated version 2. The research team made a backward translated synthetic English version by discussion. The forward and backward translations would continue until the semantic consistency rate of the backward translated version reached 95% or more with the original version (20).

#### Cultural Adaption

There were five experts in rehabilitation science, all Chinese native speakers, who worked to evaluate the first version of FOGQ-CH. The inclusion criteria for evaluators were experts who were educated with a Master's degree level or above, had at least 10 years of work experience in clinical practice, research, or education, and had professional knowledge of PD. According to their suggestion, some words were culturally adapted. For example, the words “gait difficulties” in question 2 were revised to “walking problem” because the term “walking problem” is easier to understand as a Chinese expression. After modification as per the suggestions of the experts, the second version (Pre- final Chinese version) was established.

We invited 10 Chinese patients with PD (their demographic characteristic was shown in Table 1) to complete the pre-test with the second version of FOGQ-CH so as to investigate comprehensibility and acceptability. According to their feedback, the questionnaire was revised slightly, and the final version of the FOGQ-CH was developed (see in Supplementary Material).

**Table 1:** Demographic characteristics of patients with PD, Means ± SD (minimum-maximum).

	**Pre-test study**	**Reliability study**	**Validity study**
Patients (n)	10	31	34
Sex (M/F)	6:4	13:18	18:16
Age (y)	62.80 ± 8.70 (46–76)	69.16 ± 4.45 (58–78)	67.59 ± 8.03 (46–80)
Height (m)	1.64 ± 0.07 (1.55–1.75)	1.62 ± 0.08 (1.50–1.80)	1.63 ± 0.07 (1.52–1.80)
Weight (kg)	65.00 ± 6.82 (52–75)	61.26 ± 11.51 (36–78)	59.94 ± 10.17 (36–75)
MMSE score (point)	27.40 ± 2.07 (25–30)	27.52 ± 1.84 (24–30)	28.15 ± 1.73 (24–30)
PD duration (y)	6.40 ± 6.08 (0.5–19)	7.19 ± 4.66 (2–19)	6.99 ± 4.86 (1–20)
Modified H&Y stage “ON” (stage)	1.75 ± 0.75 (1–3)	2.07 ± 0.73 (1–3)	1.93 ± 0.80 (1–4)

*PD, Parkinson's Disease; MMSE, Mini-Mental State Examination; H&Y, Hoehn and Yahr; M/F, Male/Female*.

### Part 2: Reliability and Validity Study

#### Participants

We recruited participants from Xinhua Hospital Affiliated to Shanghai Jiaotong University School of Medicine in Shanghai City and Jinhua Hospital of traditional Chinese medicine in Zhejiang Province. Thirty-one Chinese-speaking patients with PD volunteered for the reliability study, and 34 participants for the predictive study. The demographic characteristics of the participating patients with PD are summarized in Table 1. The inclusion criteria of participants were as follows: patients diagnosed with Idiopathic PD by neurologists, lasting over 6 months; Modified Hoehn and Yahr stage ≤ 4 in “off” phase according to the description of the patient; patients in stable treatment over 2 months since last modification; score on the Mini-Mental State Examination of over 24/30; and the patient could be able to walk at least 50 m with or without a walking aid. Finally, patients had symptoms of FOG as indicated by patients and their caregivers during the last week.

#### Material

The first convergent validity measurement employed was the UPDRS, as it is widely used in patients with PD. It has four parts (Parts I-IV). Part II and Part III are about ADL and Motor performance assessment. Part II has 13 questions and Part III has 14 questions, where each question has five categories scored from 0 to 4, where 0 means normal and 4 means the maximum amount of the problem.

The TUGT is a measurement tool used to evaluate the balance, posture control, and mobility of patients with PD (21). Participants are required to stand up from a chair with armrests (seat height of 50 cm, armrest height of 65 cm) (22), walk three meters as quickly and safely as possible, cross an obstacle on the floor, turn around, walk back, and sit down. The tester records the time from standing up to sitting down. For the TUGT-Cog test, the patient is asked to perform the same TUGT while counting backwards by sevens from a randomly selected number between 90 and 100.

In 10MWT, the patient is instructed to walk 14 m at a fast but safe speed. The first and last 2 m are given to accelerate and decelerate, respectively, and the 10 m zone in the middle is assessed. The time and number of steps are recorded. Walking speed is calculated by dividing the time needed by 10 m (meters/second). The step lengths were calculated by dividing steps by 10 m (meters/numbers).

### Data Collection

For the reliability study, the FOGQ-CH was filled in twice by 31 patients with PD. The interval between the two tests was 7–10 days.

For the validity studies, content validity, convergent validity, and predictive validity were evaluated. Content validity was measured by five experts using the Delphi method. In the convergent validity and predictive validity studies, all assessments were conducted during the “ON” state. The numbers of falls during the 6 months post initial assessment were recorded to determine the relationship between the scores of the FOGQ-CH and the number of falls.

### Statistical Analysis

Statistical analysis was performed with Microsoft Excel 2019 and SPSS version 25.0.

Reliability analysis included calculating internal consistency and test-retest reliability. Internal consistency was calculated by Cronbach's alpha (Cα) using each item of the FOGQ-CH. A strong internal consistency should show a moderate correlation when Cα values are between 0.70 and 0.95 (23).

For the test-retest reliability, an Intraclass Correlation Coefficient [ICC (3.1)] with two-way fixed model, single measure type, and absolute agreement definition was calculated for each item (24). ICC values were classified as poor (<0.50), moderate (between 0.50 and 0.75), good (between 0.75 and 0.90), and excellent (over 0.90) (23). With this ICC, the MDC was calculated using the following formula (25, 26):


$$\begin{array}{c} {\text{MDC}}_{0.90}\;=\;\text{SEM}\times 1.65\times \sqrt{2} \end{array}$$

$$\begin{array}{c} \text{SEM}\;=\;\text{S}\sqrt{1-IC\text{C}} \end{array}$$

$$\begin{array}{c} \%\text{MDC}\;=\;\frac{{\text{MDC}}_{0.90}}{\text{Maximum}}\times \% \end{array}$$

where SEM was the standard error of measurement, and s was the standard deviation of the measurements taken at the first time.

Content validity was tested by the item-content validity index (I-CVI) and the scale-content validity index (S-CVI). The experts rated each item in terms of its relevance to the underlying construct using a 4-point scale where 1 meant not relevant, 2 somewhat relevant, 3 quite relevant, and 4 highly relevant. The number of experts who gave a rating of 3 or 4 divided by the total number of experts for each item is calculated for the I-CVI. The I-CVIs was then added and divided by the number of items to calculate the S-CVI where I-CVI of 0.8 was considered quite relevant, and S-CVI of 0.8 or higher was acceptable (27).

Convergent validity was measured by Spearman's correlation. The correlation coefficient (*rho*) value was classified as negligible (between 0 and 0.10), weak (between 0.10 and 0.39), moderate (between 0.40 and 0.69), strong (between 0.70 and 0.89), and very strong (between 0.90 and 1.00) correlation (28). In addition, the difference between the TUGT and TUGT-Cog was determined by a Paired-Samples *t*-test.

Predictive validity was measured by the area under the receiver operating characteristic curve (AUC) in order to evaluate the ability of the FOGQ-CH and to discriminate the number of falls. Two conditions were analyzed. The first was defined as positive by one or more falls, and the second condition was defined as positive by two or more falls. A cutoff score was calculated if the AUC score was significant (*p* < 0.05). The cutoff value was calculated by the maximum value of Youden's Index, which corresponds to the maximum difference between sensitivity and 1– specificity in the receiver operating characteristic curve (ROC).

## Results

### Reliability Study

Reliability results showed a satisfactory internal consistency, with a Cα value of 0.823. Test-retest reliability results are shown in Table 2. For six items, the ICC value ranged from 0.469 to 0.848, and the total score ICC was 0.786, indicating good test-retest reliability. The FOGQ-CH's MDC_0.90_ was 4.538, and the %MDC was 18.908%.

**Table 2:** Intraclass correlation coefficient (ICC) of freezing of gait questionnaire, Chinese language version (FOGQ-CH).

**Index**	**I-CVI (*n* = 5)**	**ICC (95% CI, *n* = 31)**
Item 1	1.000	0.848 (0.710–0.924)
Item 2	0.800	0.710 (0.479–0.849)
Item 3	1.000	0.648 (0.440–0.834)
Item 4	1.000	0.679 (0.433–0.831)
Item 5	1.000	0.700 (0.467–0.842)
Item 6	1.000	0.469 (0.153–0.700)
S-CVI	0.967	
Total score		0.786 (0.578–0.894)

*ICC, Intraclass Correlation Coefficient; FOGQ-CH, Freezing of Gait Questionnaire, Chinese-language version; I-CVI, Item-Content Validity Index; S-CVI, Scale-Content Validity Index; CI, Confidence Interval*.

### Validity Study

In terms of content validity, the I-CVI for each item ranged from 0.80 to 1.00, and the S-CVI was 0.97, as shown in Table 2.

The convergent validity results showed that all PD-related measurements were moderately correlated with FOGQ-CH (Table 3). UPDRS II, UPDRS III, TUGT, TUGT-Cog, and the number of falls had a positive correlation with the FOGQ-CH. As expected, 10MWT speed and 10MWT step length showed a negative correlation with the FOGQ-CH. A significant difference (*t* = −3.390, *p* = 0.002) was found between TUGT (12.317 ± 5.433) and TUGT-Cog (15.082 ± 9.363) with the Paired-Samples *t*-Test.

**Table 3:** FOGQ-CH correlations with indices.

**Index**	**Correlation (*rho*)**	***p*-value**
UPDRS II	0.560	0.001
UPDRS III	0.451	0.007
TUGT	0.556	0.001
TUGT-Cog	0.557	0.001
10MWT-Speed	−0.478	0.004
10MWT-Step length	−0.419	0.014
The number of falls	0.346	0.045

*FOGQ, Freezing of Gait Questionnaire; UPDRS II, Unified Parkinson's Disease Rating Scale Part II; UPDRS III, Unified Parkinson's Disease Rating Scale Part III; TUGT, Timed Up and Go Test; TUGT-Cog, Timed Up and Go Test in cognitive task; 10MWT, 10-Meter Walk Test*.

At the 6 months follow-up, five patients experienced 1 fall, one patient 2 falls, two patients 3 falls, one patient 5 falls, one patient 8 falls, and one patient experienced 12 falls. In summary, there are 32.4% (11/34) patients who experienced at least one fall and 17.6% (6/34) patients who experienced two or more falls. In the first condition, one or more falls was defined as positive, AUC = 0.684 (*p* = 0.087). In the second condition, two or more falls was defined as positive, AUC = 0.777 (*p* = 0.036), and the cutoff score derived from the ROC analysis was 7.5 points, the sensitivity was 100%, and the specificity was 60.7%.

## Discussion

Freezing of Gait is one of the major motor disorders in patients with PD. It is one of the risk predictors of falling in patients with PD, and its pathophysiology is still unclear. Being older, falls, hallucinations, having anxiety, living in the countryside, having an akinetic-rigid style, and onset of lower limbs may be the predictors of FOG in Chinese patients (29, 30). The FOGQ has been found to be a reliable and valid measurement tool when used to assess FOG in other language versions (4, 5, 8–11). The questionnaire had no floor or ceiling effects, and it had a high internal consistency, good inter and intra-rater reliability, good content, and convergent validity. The Movement Disorder Society (MDS) Task Force on Rating Scales also recommended the FOGQ in evaluating FOG in 2016 (31). Accordingly, development of a Chinese version of the FOGQ is important for Chinese patients with PD, their caregivers, and clinical workers.

### Reliability Study

In the reliability study, the index of internal consistency, the Cα, for the FOGQ-CH was 0.823, a value which is similar to that found with other translated versions of the FOGQ (Spanish version: Cα = 0.81, German version: Cα = 0.83, and Brazilian version: Cα = 0.86) (7, 9, 10). However, the original English version (Cα = 0.94), the Swedish version (Cα = 0.95) and the Czech version (Cα = 0.91) had values for internal consistency that were very high (4, 5, 11). Nevertheless, all translated versions met the range requirement of 0.7–0.95, which meant that the FOGQ-CH has strong internal consistency (23).

The ICC value for each FOGQ-CH item ranged from 0.469 to 0.848, so each item showed a moderate correlation coefficient, and the FOGQ-CH total score ICC of 0.786 was close to that of the Brazilian version (ICC = 0.78) (10). Hence, it can be concluded that the Chinese version of FOGQ has good test-retest reliability.

The MDC_0.90_ of the FOGQ-CH was 4.538. The next index, %MDC, was 18.908%, which meant that 90% of patients with PD demonstrated random variation of fewer than 4.538 points in the FOGQ-CH. Therefore, if the FOGQ-CH was applied before and after an intervention, or when monitoring gait change after a certain period, a change of 4.538 points or more would be considered to be a true change. Though the %MDC_0.90_ of FOGQ-CH was high compared to some other questionnaires employed in assessments of patients with PD (Berg Balance Scale = 8.93%, Activities-specific Balance Scale = 13%, UPDRS II = 7.69%, UPDRS III = 10.19%) (25), it was lower than the New freezing gait of questionnaire (NFOGQ, MDC_0.95_ = 9.555, %MDC = 35.5%) according to the Hulzinga et al. (26). Compared the NFOGQ, the FOGQ showed greater capability to detect the change in FOG of patients with PD. The MDS Task Force on Rating Scales also classified the FOGQ as “recommended” and the NFOGQ as just “suggested” (31), which means that the FOGQ may still be preferable for FOG assessment. However, the Minimal Clinical Importance Difference (MCID), a meaningful index presenting the smallest beneficial or detrimental change for the patient (32), for the FOGQ is still unknown, so future research on the MCID is needed.

### Validity Study

In the content validity part of the study, five Chinese experts gave a high evaluation of the content validity, showing the FOGQ-CH items to be highly relevant to FOG. However, because there was no evaluation of content validity in previous translation versions, no comparison is possible.

According to the convergent validity results, the FOGQ-CH had a moderate correlation with UPDRS II (*rho* = 0.596) and UPDRS III (*rho* = 0.408), and in this, it was similar to the original English and Swedish version (4, 20).

The TUGT is an important index used to predict the balance ability of patients with PD, and poor balance can lead to falling (33). The FOGQ-CH (*rho* = 0.556) had a moderate relationship with the TUGT, which was different from the German version. The reason may be the testing pace used, the sample size or the selection criteria used. In this study, 34 patients with PD participated in the study, and the study with the translated German version had 27 patients. The Modified H&Y stage was 1–4 in the inclusion criteria in this study, but in the German translated version study, the H&Y stage range was 2–4 and the Modified H&Y stage was not specified in the inclusion criteria, so there are some differences making comparison difficult. The FOGQ is focused more on gait, while the TUGT is focused more on overall balance ability in patients with PD. Therefore, to some extent, the FOGQ could also reflect the walking balance ability of patients with PD. The TUGT-Cog (*rho* = 0.557) had a moderate relationship with the FOGQ-CH. Patients need to complete motor and cognition tasks simultaneously in TUGT-Cog, and more time is needed to process inputs when doing a dual-task. A study by Dreher and Grafman (34) showed that when people performed a dual-task, they activated the prefrontal-parietal neural network more than when performing a single task, so the TUGT-Cog was shown to be more sensitive at representing the defect in ability to carry out dual tasks (17). The time spent here on dual-task performance (15.082 ± 9.363) was longer than spent on the single motor task (12.317 ± 5.433). In addition, there was a significant difference (*p* < 0.001) between performance on the TUGT and TUGT-Cog, where the TUGT-Cog was more difficult for patients with PD.

In the 10MWT, the FOGQ-CH had a moderate correlation with speed (*rho* = −0.478) and step length (*rho* = −0.419). Gait speed and gait of patients with PD were influenced by motor impairment, plantarflexion muscle strength, and age (35). Small step length is one of the symptoms in PD, and step length could be a predictor of executive dysfunction in PD (36), with the smaller the step length, the more serious the disease. The AUC values to test the efficacy of fast gait speed in 10MWT in predicting falls for patients with PD within 6 months, but the result was not ideal (AUC = 0.256, *p* = 0.064). It was similar to the result by Paul et al. (37) in which they also did not agree in that the fast speed of the 10MWT could predict the falls in the future.

In terms of the number of falls, we found that the FOGQ-CH had only a weak correlation with fall numbers. There were 32.4% of patients with FOG who experienced at least one fall, and 17.6% had experienced two or more falls. According to the AUC results, the FOGQ-CH had only limited power to predict fallers and non-fallers; however, it had good predictive validity in discriminating multiple fallers at 6 months when the cut-off points of 8 was applied, which was similar to a study by Duncan and Earhart (38).

## Conclusion

The FOGQ-CH is a reliable and valid tool when used to assess the severity of FOG in Chinese-speaking patients with PD. The FOGQ-CH had good efficacy for detecting those Chinese patients with PD who have multiple falls in the following 6 months. Future studies are needed to explore the MCID of the FOGQ in patients with PD, and to use the FOGQ to monitor the amount of FOG change.

## Limitations

Although the FOGQ-CH study and most of the FOGQ translation study did not include a control group, it would be ideal to have a control group. The aggregate validity of FOGQ-CH was tested. However, the divergent validity has not been tested, e.g., UPDRS I. Hence, in the future, we can explore the divergent validity of FOGQ-CH. In addition, we can test the convergent validity of self-selected speed of 10MWT and FOQG-CH, and explore the predict validity of self-selected speed of 10MWT and other related indicators in predicting the fall condition of Chinese patients with PD in the future.

## Data Availability Statement

The original contributions presented in the study are included in the article/Supplementary Material, further inquiries can be directed to the corresponding author/s.

## Ethics Statement

The studies involving human participants were reviewed and approved by Scientific Research Ethics Committee of Shanghai University of Sport. The patients/participants provided their written informed consent to participate in this study.

## Author Contributions

PT and XSha collected data, analyzed data, and drafted the manuscript. JZ, ZW, YD, and XShe helped to collect the data. YG, XShu, HW, YX, and ZL helped in the translation and cultural adaptation of the FOGQ-CH. RA helped to edit the manuscript. JH participated in the study design, analyzed data, and edited the manuscript. All authors contributed to the article and approved the submitted version.

## Funding

This study was funded by Public Welfare Foundation of Science and Technology Department of Zhejiang Province (Grant No.: LGF20H170003), Public Welfare Projects of Jinhua Science and Technology Bureau (Grant No.: 2019-4-071), National Nature Science Foundation of China (Grant No.: 31870936), Ministry of Education of China (Humanities and Social Science Project, Grant No.:18YJA890006), Program of Science and Technology Commission of Shanghai Municipality [Excellent Academic Leader (Youth) of Science and Technology Innovation Action Plan, Grant No.: 20XD1423200], and Program of Shanghai Administration of Sports (Sports Science and Technology Project, Grant No.: 21Q006).

## Conflict of Interest

The authors declare that the research was conducted in the absence of any commercial or financial relationships that could be construed as a potential conflict of interest.

## Publisher's Note

All claims expressed in this article are solely those of the authors and do not necessarily represent those of their affiliated organizations, or those of the publisher, the editors and the reviewers. Any product that may be evaluated in this article, or claim that may be made by its manufacturer, is not guaranteed or endorsed by the publisher.
